# Analysis of differences in gut microbiota structure and function in Bactrian camels under different breeding patterns from northern and southern Xinjiang, China

**DOI:** 10.3389/fvets.2026.1892252

**Published:** 2026-08-31

**Authors:** Huaibing Yao, Aerman Haire, Zhi Wang, Tongxuan Ge, Yongxuan Liu, Min Hou, Weidong Cui

**Affiliations:** 1 Institute of Microbiology, Academy of Agricultural Sciences of Xinjiang Uyghur Autonomous Region, Urumqi, China; 2 Xinjiang Agricultural Vocational and Technical University, Changji, China; 3 Management Committee of Engebei Ecological Demonstration Zone, Ordos, China

**Keywords:** Bactrian camel, carbohydrate-active enzymes, gut microbiota, metagenomic sequencing, northern and southern Xinjiang

## Abstract

**Introduction:**

The lactating Xinjiang Junggar camel is a major dairy breed with excellent milk production performance. In northern Xinjiang, camels are mainly reared under traditional grazing, while those in southern Xinjiang are raised under barn feeding due to grassland scarcity.

**Methods:**

Nine lactating camels aged 6–7 years old and nine juvenile camels aged 8 months old were selected from each region. Fresh fecal samples were collected from the rectum of each camel for metagenomic sequencing analysis.

**Results:**

The findings indicated that the diversity and abundance of gut microbiota in the BJT group were significantly increased compared with those in the NJT group. Furthermore, the diversity and abundance of gut microbiota in adult lactating camels from both regions were superior to those in juvenile camels. Among the gut microbiota, Actinomycetota, Campylobacterota, Ascomycota, and Thermoplasmatota presented significantly higher relative abundance in the BJT group than in the NJT group (*p* < 0.05). The relative abundance of *Acinetobacter* and *Halomonas* was also significantly elevated in the BJT group, whereas *Escherichia* and *Prevotella* were significantly less abundant. Furthermore, the relative abundance of *Bifidobacterium* and *Lactobacillus* declined significantly in adult lactating camels compared to that in juvenile camels. There were notable differences in the functional annotations of metabolic pathways, suggesting that the gut microbiota in the BJT group was primarily engaged in nitrogen cycling, decomposition of various organic compounds, and fatty acid metabolism, which were linked to nutrient metabolism, detoxification, and biosynthesis. In contrast, the gut microbiota in the NJT group was associated with such functions as cell recognition, signal transduction, and immune response. Glycoside Hydrolases and Glycosyltransferases constituted the primary carbohydrate-active enzymes within the gut microbiota in Bactrian camels, and they were conducive to the degradation of plant cellulose.

**Discussion:**

Since the intestines of juvenile camel are in the developmental stage, the genera *Bifidobacterium* and *Lactobacillus* potentially play significant roles in intestinal maturation. Notably, the gut microbiota in Bactrian camels from northern Xinjiang are characterized by high relative abundance of microorganisms that are halophilic and involved in crude fiber degradation. In contrast, Bactrian camels under barn feeding in southern Xinjiang exhibit higher abundance of the genera *Escherichia* and *Prevotella*, implying geographical and feeding conditions are associated with the variations in gut microbiota variations.

## 1 Introduction

Bactrian camels (*Camelus bactrianus*) are indispensable livestock species in the arid regions of northwest China, with remarkable adaptability to extreme environmental conditions (drought, high temperature, low temperature, etc.) and robust immune characteristics, so they can survive and maintain stable reproduction in harsh desert and Gobi ecosystems (1). Researchers have classified Bactrian camels into two subtypes, Junggar (Northern Xinjiang) and Tarim (Southern Xinjiang), based on their geographical distribution, morphological traits, and body size. The milk-producing Junggar Bactrian camels are recognized as the dominant dairy breed due to their superior lactation performance, becoming a core resource promoting the development of camel milk industry in Xinjiang (2–4).

However, low milk yields and prolonged breeding cycles of Bactrian camels lead to a supply–demand imbalance in the camel milk market, thus requiring frequent cross-regional trading and import (5). To enhance milk yields, enterprises and herders in southern Xinjiang, China, have extensively sourced Junggar Bactrian camels from northern Xinjiang for breeding purposes. In northern Xinjiang, herders and enterprises predominantly employ traditional extensive grazing systems for animal husbandry, which are dependent on natural pasture vegetation. Such a long-term grazing pattern not only fosters strong resistance to diverse pathogens in camels, but also shapes a unique gut microbial ecosystem adaptable to natural forage. However, the limited pasture availability in southern Xinjiang drives livestock producers to shift to intensive barn-feeding systems for camel husbandry, which are drastically different from the grazing system in terms of diet composition, feeding frequency, and living environment of camels, thus inevitably affecting the structure and function of the gut microbiota.

It is widely recognized that food in camels is digested mainly through gut microbial fermentation (6), and the fecal microbial composition exhibits seasonal dynamics. Specifically, the microbial diversity increases during warm seasons, while the abundance of fiber-degrading bacteria increases in cold seasons (7). Under the same environmental conditions, microbial diversity is significantly higher in adult female camels than in suckling calves, indicating that growth stage is also a key factor affecting gut microbial characteristics (8). Despite these existing findings, there is a significant lack of feeds formulated with scientifically optimized formulations tailored to the digestive characteristics of Junggar Bactrian camels in different regions of Xinjiang. This gap has directly led to substantial operational challenges in practical husbandry, including suboptimal feeding efficiency, nutritional imbalances, and even potential health risks caused by microbial dysbiosis.

The gut microbiota in camels comprises bacteria, protozoa, archaea, and fungi, directly influencing camels’ physiological functions, metabolic homeostasis, and health status. Notably, dietary composition under different husbandry systems serves as a direct determinant of gut microbiota structure in camels. Previous studies primarily focused on breed characteristics, milk composition, and husbandry techniques (9, 10). However, few systematic studies have been conducted on the differences in the gut microbiota in Bactrian camels at distinct growth stages from southern and northern Xinjiang under various breeding patterns. The scarcity of such studies has hindered the elucidation of mechanisms through which the camels adapt to diverse breeding patterns and impeded the formulation of targeted feeding strategies. This study took the lead in exploring the disparities in specific breeding patterns between southern and northern Xinjiang, China. Targeting such issues as suboptimal feed efficiency and nutritional imbalances, it thoroughly elucidated the relationships among gut microbiota structure, functional potential, and environmental adaptability in Bactrian camels. The findings are expected to provide a theoretical foundation for the optimization of intensive camel breeding practices and the development of region-specific scientific feeding regimens.

## 2 Materials and methods

### 2.1 Study area and animal selection

This study was conducted in two distinct regions of Xinjiang, China: In northern Xinjiang (BJT group), Fuhai County, Altay Prefecture (47°1′20″N, 87°13′7″E) was selected, which has a temperate continental climate with a mean annual temperature of 4.5 °C and annual precipitation of 150–200 mm. In southern Xinjiang (NJT group), Keping County, Aksu Prefecture (40°32′40″N, 79°1′44″E) was selected, which has an arid desert climate with a mean annual temperature of 11.2 °C and annual precipitation of 50–80 mm. A total of 36 healthy Bactrian camels were selected from multiple local herds that were mutually independent, with the inclusion criteria strictly followed to ensure homogeneity of physiological status and minimize confounding factors. The cohort was divided into two age groups based on developmental stage and production purpose: One consisted of adult lactating camels (*n* = 18, 6–7 years old). As the reproductive adaptation period for Bactrian camels typically concludes by the age of 5–6 years old, the camels have fully matured body at this age. Clinical examinations confirmed that these camels were free of diarrhea and other digestive disturbances. The camels in this group exhibited stable lactation performance (a period of 90–120 days postpartum) and had no past history of metabolic or gastrointestinal disorders (verified by veterinary examination). Fully developed gut microbiota and organ systems can guarantee consistent milk production, so the camels in this group possess the optimal reproductive performance in pastoral systems. The other comprised juvenile camels (*n* = 18, approximately 8 months old). This critical developmental window marks the transition from exclusive milk consumption to mixed feeding (solid forage intake initiated at 3–4 months old, and weaning completed by 8–10 months old). Moreover, the gastrointestinal tract undergoes rapid morphological and functional maturation during this period, including gut papillation development and establishment of adult-like microbial communities.

### 2.2 Feeding management

The lactating Bactrian camels in northern Xinjiang were primarily raised under traditional free-range grazing. The main forage species included plants from the Chenopodiaceae, Poaceae, and Fabaceae families. The camels were provided with water at noon and in the evening. In southern Xinjiang, the lactating Bactrian camels were raised in confinement, with a forage primarily made of corn, silage, alfalfa, and cottonseed cake. They were allowed to eat forage and drink water freely at scheduled times. The composition and nutritional levels of the basal diet are shown in Table 1. The nutritional composition (crude protein, neutral detergent fiber, acid detergent fiber and crude ash) of major Chenopodiaceae and Poaceae forages provided for the grazing camels during mid-September is summarized in Table 2. The same feeding methods were adopted for juvenile camels in both southern and northern Xinjiang. Before 3 months of age, the juvenile camels mainly fed on mother camel milk. After 3 months of age, they were raised in barns and supplemented with a small amount of alfalfa and flaxseed cake. After 1 year of age, the camels were weaned. Fresh fecal samples were collected around mid-September. All selected camels were not treated with any antibiotics or probiotic supplements within at least 2 months prior to sampling to avoid interference with gut microbiota. Samples were collected from fasted animals (no food intake for 10 h) at approximately 07:30, prior to morning feeding. Rectal fecal samples from Bactrian camels in both regions were immediately placed in liquid nitrogen, transported to the laboratory, and stored at −80 °C. All animal procedures were approved by the Experimental Animal Welfare Ethics Committee of Xinjiang Agricultural University (Approval No. 2023017).

**Table 1:** Nutrient composition and concentrations of the basal diet for lactating Bactrian camels in southern Xinjiang (dry matter basis).

Raw materials	Component, %	Nutritional component	Level
Corn	10.0	Dry matter, %	65.72
Ensilage fermentation	35.0	Crude protein, %	13.6
Cotton cake	9.0	Total fiber, %	27.12
Alfalfa hay	45.0	Calcium, %	0.32
NaCl	1.0	Phosphorus, %	0.30
Total	100	Net energy intake, NEI/(MJ/kg)	5.42

**Table 2:** Estimated dry-matter nutritional composition of dominant forages consumed by free-grazing Bactrian camels in Northern Xinjiang during mid-September (dry matter basis).

Forage family	Crude protein (CP)/%	Neutral detergent fiber (NDF)/%	Acid detergent fiber (ADF)/%	Ash/%	Dietary proportion/%
*Chenopodiaceae*	10.6 ± 1.45	42.8 ± 3.12	25.1 ± 2.78	16.3 ± 2.64	60% ~ 70%
*Poaceae*	7.2 ± 1.31	57.6 ± 4.03	33.2 ± 3.25	7.4 ± 1.42	30% ~ 40%
Overall nutritional intake level	9.3 ± 1.62	49.2 ± 3.76	29.5 ± 3.01	12.5 ± 2.18	—

All nutritional values are derived from measurements of mature desert forages in the Junggar Basin, Northern Xinjiang, during mid-September.

### 2.3 Metagenome sequencing and analysis

DNA was extracted from fecal samples using the PowerFecal™ DNA Extraction Kit (Qiagen, Germany, version number: 12830–50). DNA concentration and integrity were assessed through 1% agarose gel electrophoresis using a NanoDrop 2000 spectrophotometer (Thermo Fisher Scientific, USA). Qualified DNA samples were sent to Wuhan Bena Technology Co., Ltd. for library construction and high-throughput metagenomic sequencing. Libraries were constructed according to the method and workflow specified in the TruSeq DNA Sample Preparation Guide (Illumina, 15026486 Rev. C): (1) DNA fragmentation; (2) end repair; (3) A-tailing; (4) adapter ligation; (5) fragment selection using an automated gel cutter to recover target fragments; (6) PCR enrichment of target fragments. Preliminary quantification was performed using Qubit 3.0, and libraries were diluted to 1 ng/μL. After determination of the insert size of the library using Agilent 2100, Q-PCR was performed using a Bio-RAD CFX 96 real-time PCR system and Bio-RAD KIT iQ SYBR GRN to accurately quantify the effective library concentration (>10 nmol/L). Then the NovaSeq X Plus platform was employed for paired-end sequencing of the qualified libraries. Raw sequencing data were processed by fastp v0.20.1 software to remove adapters and low-quality sequences (11), thereby yielding clean reads. Genome assembly of the sequencing data was performed using MEGAHIT v1.1.3 software based on the iterative k-mer De Bruijn graph (DBG) method (12). Gene calling and annotation were performed using Prodigal v2.6.3 (13), and open reading frames (ORFs) were predicted. To eliminate analytical bias caused by the large discrepancy in sequencing depth between grazing camels (BJAT and BJYT) and captive camels (NJAT and NJYT), all samples were rarefied to the minimum clean read count of all datasets prior to non-redundant gene set construction, gene quantification and alpha diversity calculation. Rarefaction was performed to standardize total sequencing depth across samples and mitigate distortions in alpha diversity metrics originating from unequal sequencing effort. However, rarefied raw reads do not account for variation in gene length and are inappropriate for comparison of gene abundance across samples. Therefore, gene abundance was further normalized to transcripts per million (TPM) after rarefaction. TPM aimed to correct gene-length bias and generate normalized values suitable for intersample comparison. This two-step normalization strategy sequentially addresses sequencing depth heterogeneity and gene length variation. A potential drawback of rarefaction is the loss of partial sequencing data, which may lower the detection power for low-abundance genes. Quantification of the non-redundant gene set was performed using Salmon v0.13.1 software, with gene abundance normalized using the TPM algorithm (14). After that, taxonomic and functional analyses were performed using the normalized abundance profiles. The data have been uploaded to the NCBI Sequence Read Archive (SRA) database under accession number PRJNA1199154.

### 2.4 Bioinformatics analysis

DIAMOND was employed to align the non-redundant gene set with gene databases such as KEGG and Carbohydrate-Active Enzymes (CAZy), so as to obtain the information on species and functional annotations (15). The abundance of each species was calculated based on the sum of corresponding gene abundance, and the abundance of species in each sample was statistically analyzed at various taxonomic levels. Annotations for carbohydrate-active enzymes were generated using HMMER v3 against the dbCAN database (16). QIIME2 v2020.6 was used to evaluate the alpha and beta diversity of the samples (17). SPSS v24.0 was employed to perform non-parametric Kruskal-Wallis rank-sum tests to analyze differences between groups at the microbial phylum and genus levels (18). To avoid false positives caused by massive parallel comparisons in metagenomic sequencing data, the Benjamini–Hochberg method for false discovery rate (FDR) control was applied for multiple-testing correction. All *p*-values presented in Figures were FDR-adjusted, and an adjusted *p*-value < 0.05 was considered of statistical significance. Composition analyses and non-metric multidimensional scaling (NMDS) of the gut microbiota were performed in R version 3.4.0, and related plots were generated (19). QIIME2 was used to test for significant differences in the enriched biological functions of the gut microbiome across different groups (20).

## 3 Results

### 3.1 Metagenomic sequencing results

Microbial metagenomic sequencing was performed on fresh fecal samples from the BJT group (BJAT1–BJAT9 and BJYT1–BJYT9) and the NJT group (NJAT1–NJAT9 and NJYT1–NJYT9). A total of 491.4 Gb of raw data were generated, with an average of 13.65 Gb per sample. As shown in Table 3, each sample assembly demonstrated high quality.

**Table 3:** Sample sequencing and assembly results.

Sample	‌Clean reads	GC (%)	GC (%)	GC (%)	N50 (bp)
BJAT1	30831319	43.25	98.51	95.13	1278
BJAT2	31629236	44.67	98.15	94.15	1044
BJAT3	30929855	44.55	98.47	95.05	1037
BJAT4	31734625	44.15	98.32	94.26	1135
BJAT5	31627931	44.32	98.24	95.08	1024
BJAT6	30528845	44.42	98.38	95.36	1114
BJAT7	30674862	44.14	98.42	95.05	1037
BJAT8	32681510	44.33	98.72	95.08	1125
BJAT9	29235254	44.43	98.21	95.29	1223
BJYT1	29155124	43.94	98.70	95.64	1041
BJYT2	30774462	43.90	98.55	95.20	1323
BJYT3	34181511	44.77	98.29	94.50	1115
BJYT4	29235254	44.23	98.68	95.62	1185
BJYT5	31472324	43.87	98.31	94.34	1421
BJYT6	32683861	43.39	98.29	94.31	1213
BJYT7	27385257	44.36	98.54	95.52	1087
BJYT8	32452426	43.45	98.41	94.63	1324
BJYT9	32684855	43.41	98.70	94.62	1242
NJAT1	57461531	50.58	98.83	96.24	952
NJAT2	59864049	51.65	98.65	95.74	955
NJAT3	58833297	50.77	98.70	95.91	967
NJAT4	58390531	50.38	98.39	95.23	938
NJAT5	59434036	51.18	98.45	95.34	964
NJAT6	58734178	51.11	98.81	96.65	979
NJAT7	58693421	50.29	98.45	95.51	948
NJAT8	59831137	51.45	98.32	95.38	966
NJAT9	58264277	51.38	98.23	96.72	969
NJYT1	56729352	49.45	98.68	96.53	1090
NJYT2	60664329	49.15	98.87	96.41	1026
NJYT3	64015723	50.09	98.66	95.81	1079
NJYT4	58346656	50.46	98.37	95.61	1025
NJYT5	61597224	50.54	98.45	95.34	1131
NJYT6	59739632	51.21	97.97	96.46	1251
NJYT7	59446475	50.39	98.30	95.89	1021
NJYT8	62496322	50.48	98.57	95.67	1129
NJYT9	59818431	51.61	97.83	96.81	1147

### 3.2 Analysis results of alpha and beta diversity of gut microbiota in Bactrian camels

**Figure 1:**
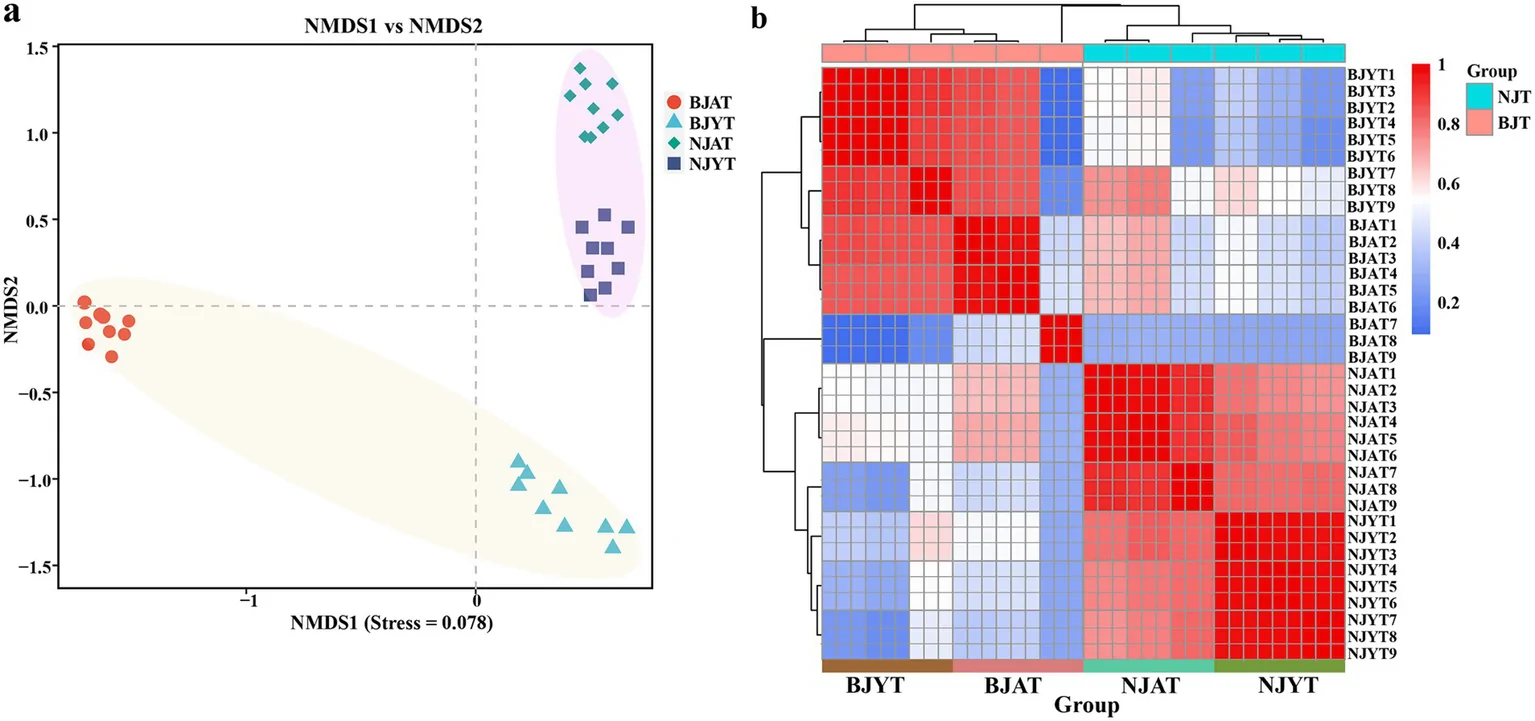
Inter-sample variation and correlation profiling results: **(a)** NMDS, non-metric multidimensional scaling (NMDS) analysis and **(b)** Heatmap of correlation analysis between samples.

As shown in Table 4, the diversity indices Shannon, Simpson, Feature, ACE, and Chao1 of the gut microbiota were lower in the NJT group than in the BJT group, as well as lower in juvenile camels than in adult lactating camels. Besides, adult lactating camels exhibited increases in gut microbiota diversity and abundance compared with juvenile camels, while the grazing camels showed greater diversity and abundance than the captive camels. NMDS analysis (Figure 1a) and correlation heatmap analysis (Figure 1b) revealed that the relatively narrow intra-group sample distances resulted in distinct clustering within each group. The wider inter-group distances signified marked differences in gut microbiota composition between the NJT and BJT groups. The NMDS stress value was 0.0784 (<0.2), suggesting a good fit and high reliability of the ordination. Correlation analysis showed significant differences among all groups, and the experimental samples exhibited excellent reproducibility.

**Table 4:** Alpha diversity analysis of the intestinal microbial community in Bactrian camels.

Item	BJAT	BJYT	NJAT	NJYT
Shannon index	5.82 ± 0.09^a^	4.42 ± 0.08^b^	4.01 ± 0.01^c^	3.53 ± 0.16^d^
Simpson index	0.95 ± 0.01^a^	0.83 ± 0.03^b^	0.80 ± 0.01^c^	0.73 ± 0.01^d^
Feature	1965.33 ± 31.05^a^	1161.50 ± 30.50^b^	1250.00 ± 18.28^c^	812.14 ± 45.25^d^
ACE index	1875.52 ± 22.05^a^	1180.40 ± 31.27^b^	1015.00 ± 18.17^c^	763.51 ± 43.12^d^
Chao1 index	1765.33 ± 31.05^a^	1091.50 ± 33.31^b^	949.01 ± 18.05^c^	627.32 ± 26.21^d^

Significant differences (*p* < 0.05) among different groups are indicated with different superscript letters.

### 3.3 Structure analysis results of gut microbiota in Bactrian camels

**Figure 2:**
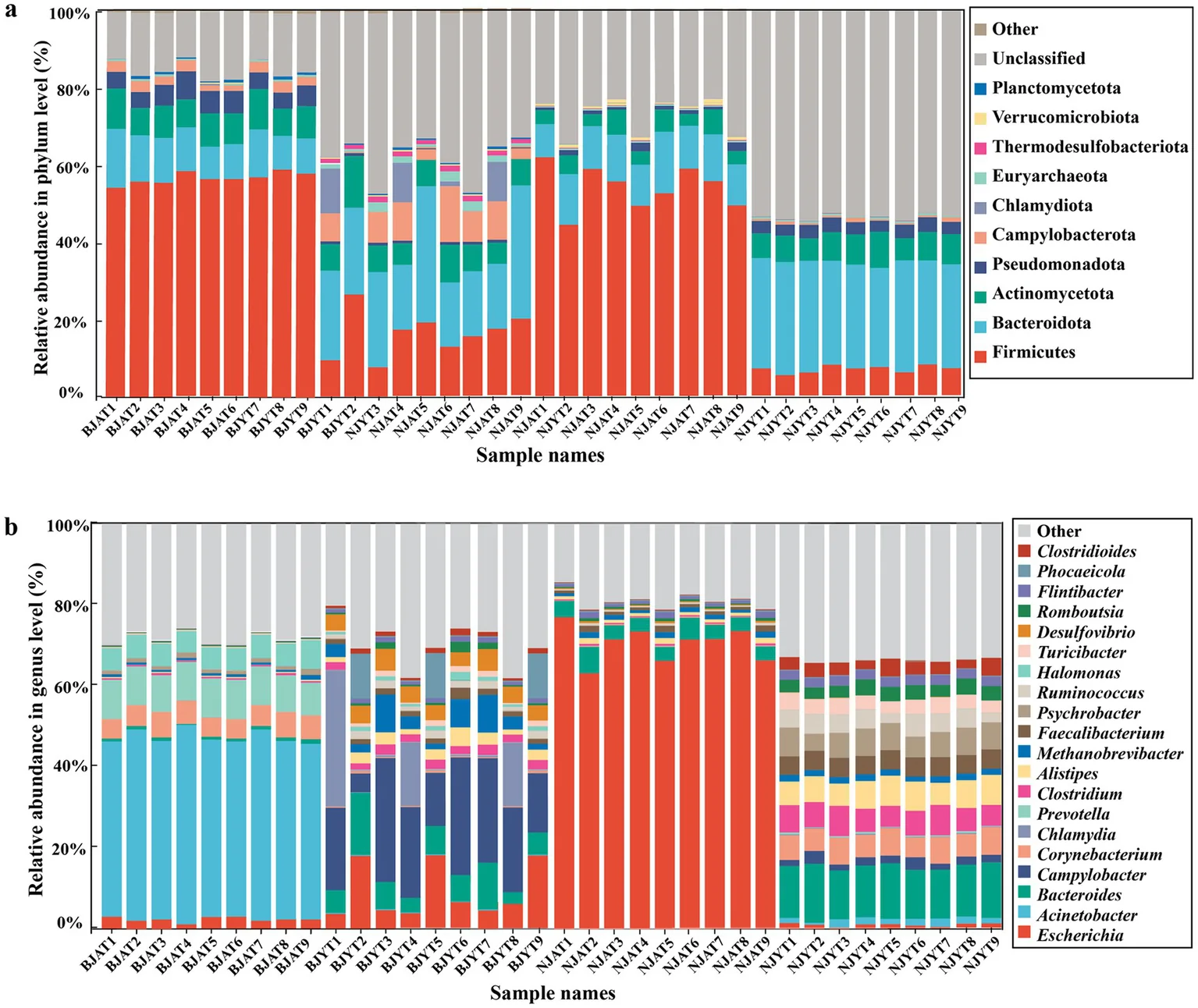
Relative abundance bar chart of intestinal microbiota species in Bactrian camels. **(a)** Phylum-level community bar chart; **(b)** Genus-level community bar chart.

The microbial community in the fecal samples from Bactrian camels comprised 4 kingdoms, 50 phyla, 16 classes, 180 orders, 400 families, and 1,065 genera. At the phylum level (Figure 2a), Firmicutes, Bacteroidota, and Actinomycetota were dominant in both BJT and NJT groups. However, the relative abundance of Pseudomonadota in BJAT camels was higher than that in NJAT camels. The relative abundance of Campylobacterota and Euryarchaeota was slightly higher, while that of Pseudomonadota was slightly lower in BJYT camels than in NJYT camels. At the genus level (Figure 2b), *Acinetobacter* was the most abundant in BJAT camels, followed by *Pseudomonas*, *Corynebacterium*, and *Halomonas*. In NJAT camels, the dominant genus was *Escherichia*, followed by *Bacteroides* and *Methanobrevibacter*. In BJYT camels, *Bacteroides*, *Campylobacter*, and *Escherichia* exhibited relatively high abundance, whereas in NJYT camels, *Bacteroides*, *Corynebacterium*, and *Clostridium* were the predominant gut microbiota.

### 3.4 Analysis results of differences in gut microbiota between the BJT and NJT groups

**Figure 3:**
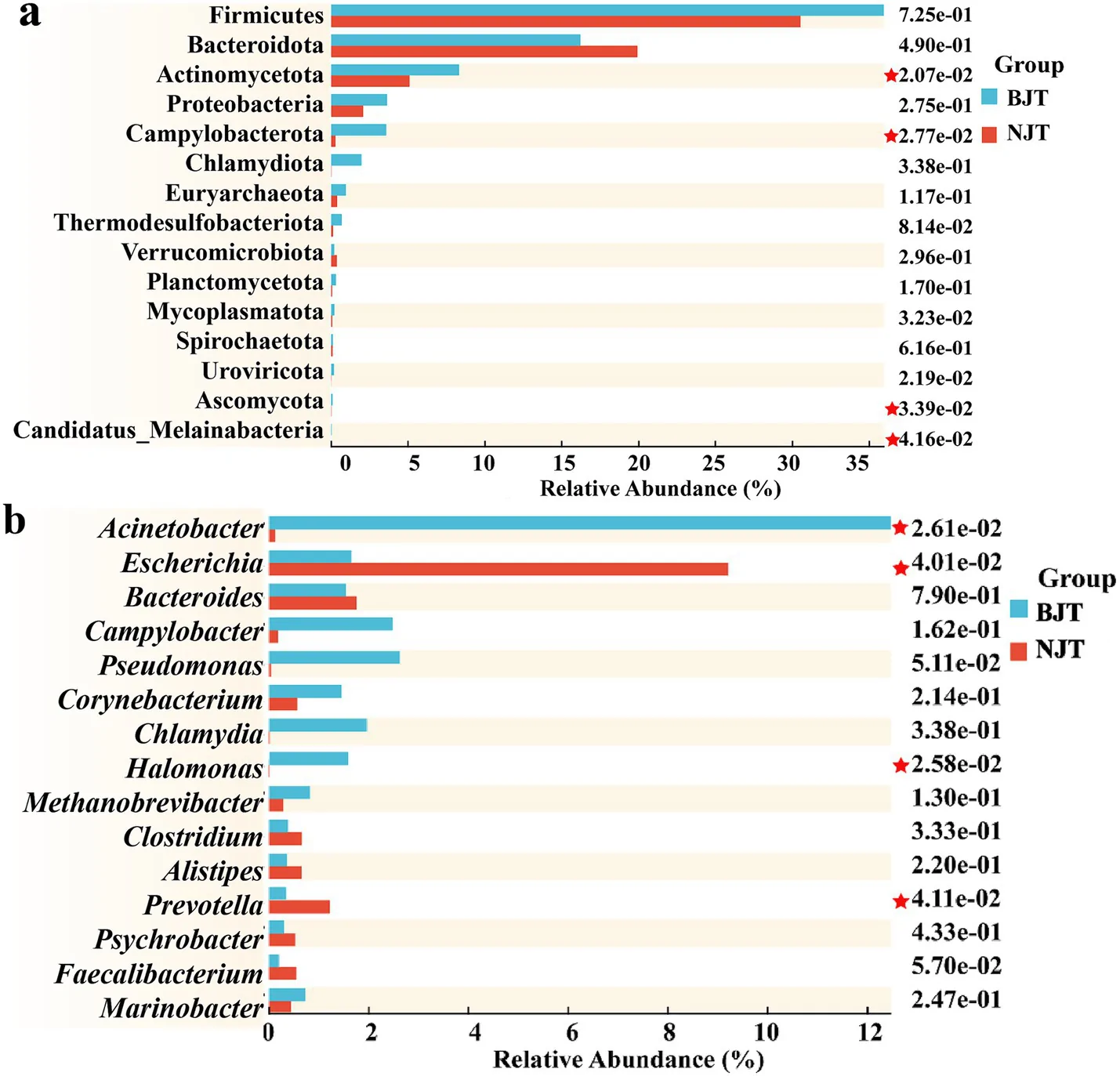
Analysis of variance between groups at the phylum and genus levels in Bactrian camels from southern and northern Xinjiang. **(a)** Bar chart of differential testing at the phylum level; **(b)** Bar chart of differential testing at the genus level. * indicates significant difference between the two groups (*p* < 0.05), **indicates significant difference between the two groups (*p* < 0.01); ***indicates significant difference between the two groups (*p* < 0.001). BJT: Northern Xinjiang grazing group, NJT: Southern Xinjiang captive group. The same applies to the figures below.

ANOVA results (Figure 3) indicated that there were differences in gut microbiota abundance between the BJT and NJT groups. Among the top 15 phyla in relative abundance, Actinomycetota, Campylobacterota, Ascomycota, and Candidatus_Thermoplasmatota in the BJT group presented significantly higher abundance than those in the NJT group (*p* < 0.05) (Figure 3a). Regarding the top 15 genera in relative abundance, *Acinetobacter* and *Halomonas* in the BJT group had significantly increased abundance compared with those in the NJT group (*p* < 0.05), while *Escherichia* and *Prevotella* showed significantly decreased abundance contrasted with those in the NJT group (*p* < 0.05) (Figure 3b).

### 3.5 Analysis results of differences in gut microbiota between adult and juvenile camels

**Figure 4:**
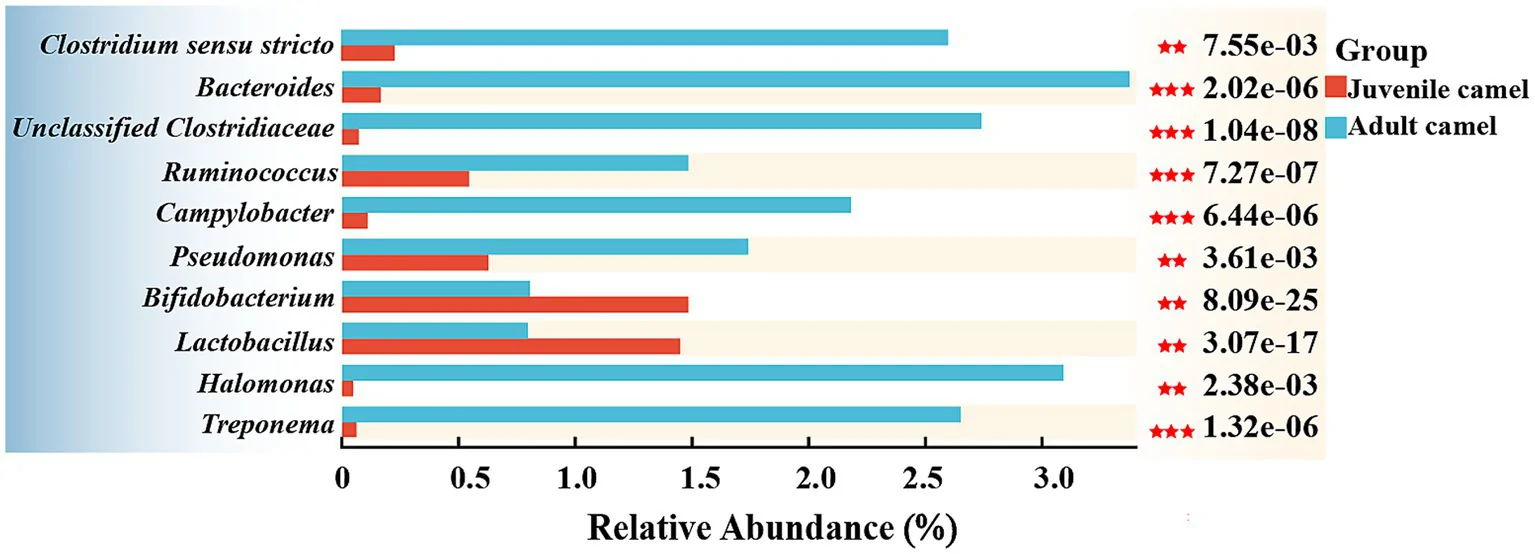
Analysis of variance between groups at the genus level for adult and juvenile camels. BJT: Northern Xinjiang grazing group, NJT: Southern Xinjiang captive group.

As for the top 10 genera exhibiting significant differences between adult female camels and juvenile camels from both regions (Figure 4), the relative abundance of *Clostridium sensu stricto*, *Bacteroides*, *unclassified Clostridiaceae*, *Ruminococcus*, *Campylobacter*, *Pseudomonas*, *Halomonas*, and *Treponema* in the fecal samples from adult lactating camels was higher than that in juvenile camels (*p* < 0.05), whereas *Bifidobacterium* and *Lactobacillus* showed lower relative abundance in adult lactating camels than in juvenile camels (*p* < 0.05).

### 3.6 Analysis results of metabolic functions of gut microbiota in Bactrian camels

**Figure 5:**
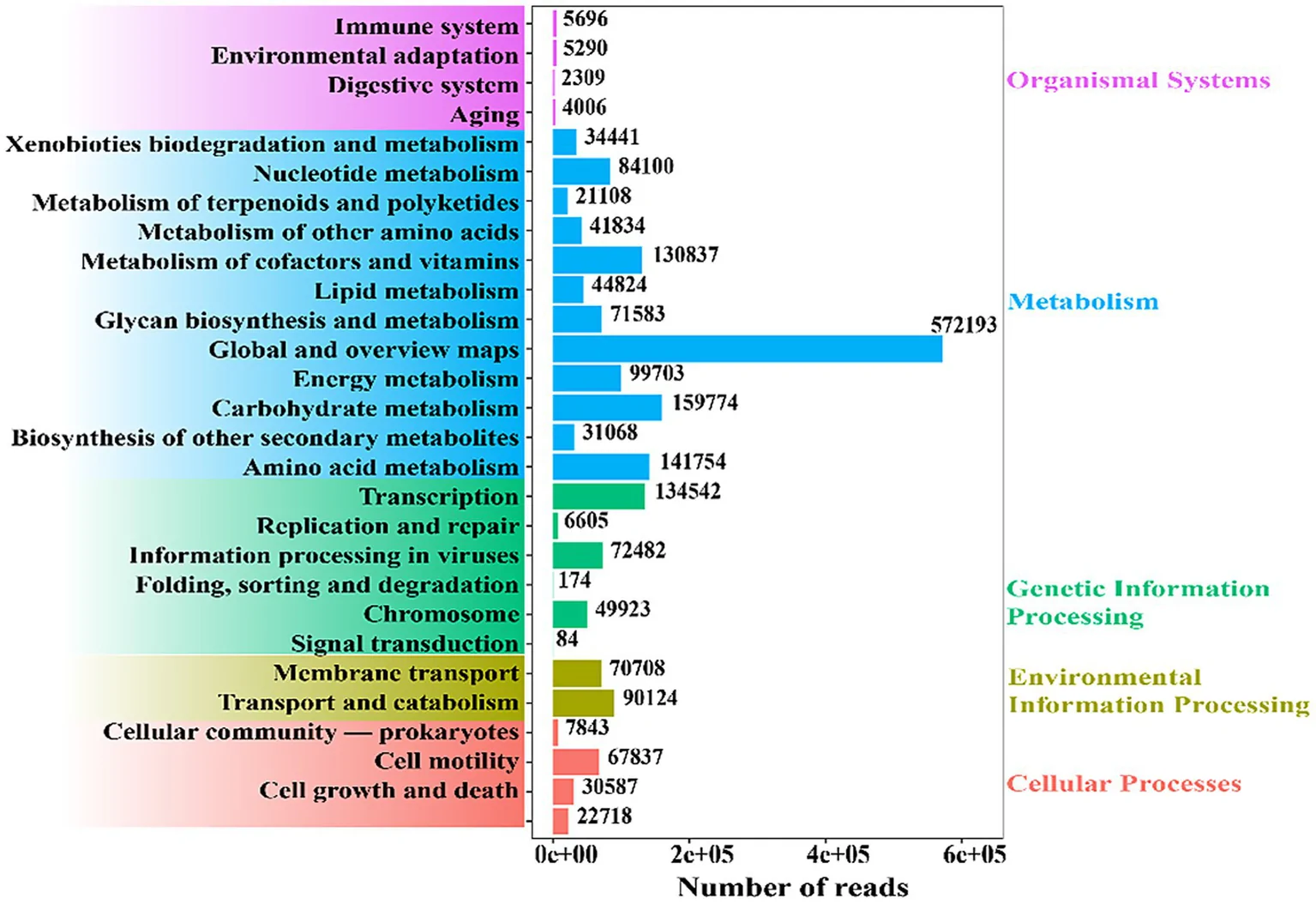
Functional prediction of Bactrian camel gut microbiome.

**Figure 6:**
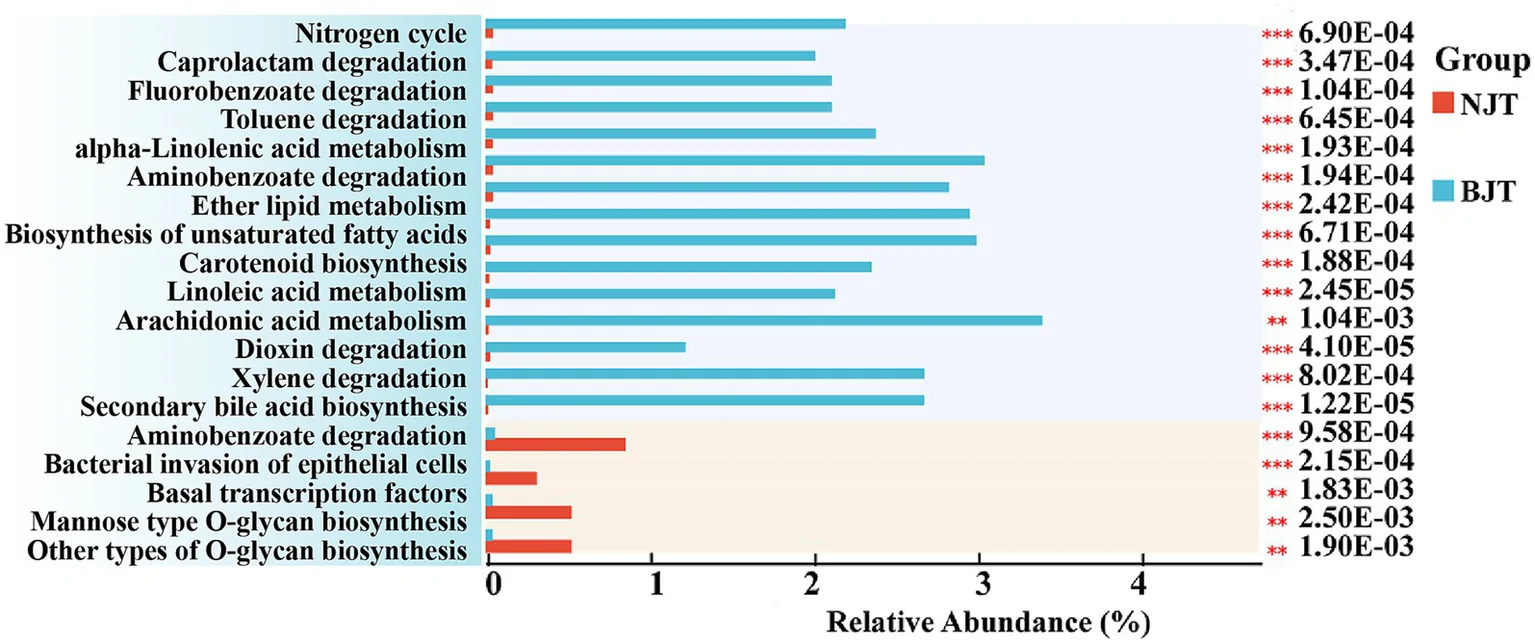
Analysis of functional differences in gut microbiota of Bactrian camels in northern and southern Xinjiang.

KEGG Level-1 functional profiling revealed that the non-redundant genes were predominantly mapped to metabolic pathways, taking up the highest proportion of sequence reads. Specifically, the global and overview map exhibited the highest abundance, followed by carbohydrate metabolism, amino acid metabolism, cofactor and vitamin metabolism, and energy metabolism. Differential analysis of the classified metabolic pathways was performed based on the functional prediction results. As shown in Figure 5, the abundance of nitrogen cycling, caprolactam degradation, fluorobenzoate degradation, toluene degradation, aminobenzoate degradation, dioxin degradation, xylene degradation, *α*-linolenic acid metabolism, ether lipid metabolism, linoleic acid metabolism, arachidonic acid metabolism, unsaturated fatty acids, secondary bile acid biosynthesis, and carotenoid biosynthesis in the intestines of BJT group were enhanced by comparison to that in the intestines of the NJT group (*p* < 0.01). In contrast, the bacterial invasion frequency of epithelial cells and the biosynthesis of mannose-type and other O-glycans in the intestines of the NJT group were raised compared with those of the BJT group (*p* < 0.01) (Figure 6).

### 3.7 CAZy-based functional analysis

**Figure 7:**
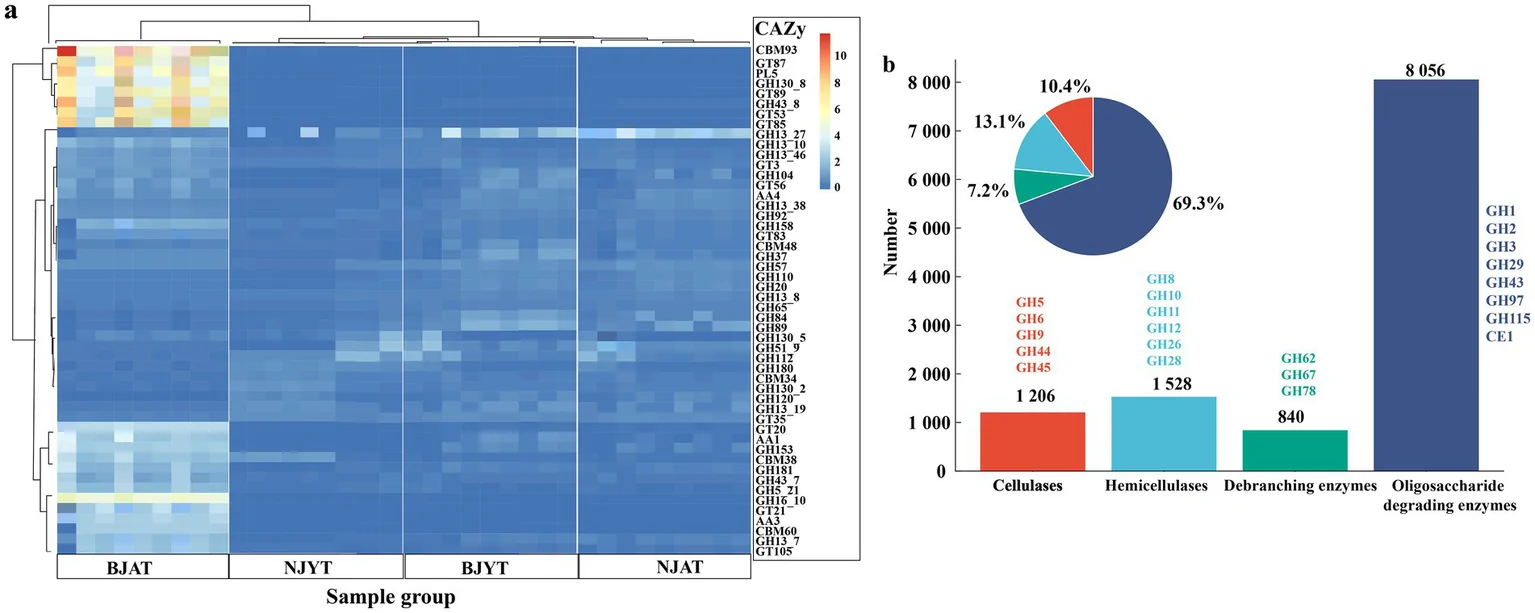
CAZy-based functional analysis. **(a)** Heatmap of CAZy enzymes in the sample; **(b)** histogram of carbohydrate active enzyme gene number annotated based on CAZy database.

The CAZy database annotated six CAZy families: Glycoside Hydrolases (GHs) (55.16%), Glycosyltransferases (GTs) (26.04%), Carbohydrate Esterases (CEs) (13.26%), Carbohydrate-Binding Modules (CBMs) (3.87%), Polysaccharide Lyases (PLs) (1.21%), and Auxiliary Activities (AAs) (0.46%), indicating that the gut microbiota obtained from Bactrian camels in both regions possesses a complete plant cellulose degradation system. Functional annotation of CAZy families was performed on samples collected from Bactrian camels in both regions, and a total of 474 CAZy families were identified. As shown in Figure 7a, GHs and GTs were the main CAZy families involved in plant cellulose degradation in the gut microbiota of Bactrian camels. The CAZy families exhibited significant differences in relative abundance among adult lactating camels from northern Xinjiang, their calves, and camels from southern Xinjiang, with CBM93, GT87, PL5, GH130, GT89, GH43, GT53, and GT85 presenting higher relative abundance. As shown in Figure 7b for the distribution of GHs within the gut microbita in Bactrian camels, oligosaccharide-degrading enzymes were the most abundant (69.3%) class of carbohydrate metabolism enzymes, followed by hemicellulases (13.1%), cellulases (10.4%), and debranching enzymes (7.2%). These results indicated the importance of these GH enzymes to the degradation of carbohydrate components of plant cell wall materials in the rumen of camels.

## 4 Discussion

The gut microbiota in ruminants constitute a complex and dynamic ecosystem. As a regionally distributed specialty livestock, camels rely more on gut microbial fermentation for digestion, and their strong resistance against stress and tolerance to coarse feed may be associated with the gut microbiota. Gut microorganisms colonize in the host through external pathways and can produce various small-molecule metabolites (e.g., short-chain fatty acids) that directly or indirectly participate in and regulate the host’s metabolism and immune response via the enteric nervous system and peripheral circulatory system. Therefore, the gut microbiota coexists interdependently inside the host and undergoes dynamic changes in response to alterations in the living environment.

For domestic Bactrian camels from different regions, Chinese researchers have found that Firmicutes, Bacteroidota, and Pseudomonadota serve as the core gut microbiota in adult Bactrian camels from Balikun County, Jeminay County, and Inner Mongolia (21, 22). Both Firmicutes and Bacteroidota exhibit strong adhesion to neutral detergent fiber in forage. Firmicutes are closely associated with the absorption of fatty acids (21), while Bacteroidota is primarily implicated in carbohydrate metabolism (23). Pseudomonadota are related to host immunity and metabolic functions (24, 25). The characteristic gut microbiota in camels at the phylum level is susceptible to environmental conditions. Lavrentyeva et al. (26) demonstrated that different grazing conditions significantly affect the characteristic gut microbiota in dromedary camels. Verrucomicrobia is a characteristic phylum in fecal samples under free-grazing and mixed-grazing conditions, while Actinomycetota is a characteristic phylum under captive conditions (27). Although core microbiota are predominant in the intestinal tract of camels, external breeding conditions impose striking shifts in gut microbiota within healthy camels at the phylum level. The drastic reduction of structural fiber intake under captive conditions may drive remarkable variation in dominant phyla such as Actinomycetota and Campylobacterota, thereby profoundly altering the composition of characteristic microbiota. These correlative observations imply that dietary fiber level may act as a key driver shaping the structure of gut microbiota in Bactrian camels at the phylum level, while causal evidence still needs verification via controlled feeding experiments.

At the genus level, dominant/subdominant taxa had their own peculiarities among various groups of study animals. *Acinetobacter* was dominant in grazing BJAT camels, followed by *Monas*, *Corynebacterium*, and *Halomonas*. Conversely, *Escherichia*, *Bacteroides* and *Methanobrevibacter* were the most abundant in captive NJAT camels. As for the gut microbiota in BJYT camels, *Bacteroides*, *Campylobacter*, and *Escherichia* showed the highest relative abundance, while *Bacteroides*, *Corynebacterium*, and *Clostridium* constituted the predominant gut microbiota in NJYT camels. Among the gut microbiota in juvenile camels from both BJYT and NJYT, *Bacteroides* had significantly close correlations with their nutritional metabolism and immune functions. According to the results of differential analysis, the BJT group had significantly higher abundance of *Acinetobacter* and *Halomonas* as well as significantly lower abundance of *Escherichia* and *Prevotella* than the NJT group. It is noteworthy that, the high relative abundance of *Acinetobacter* in grazing camels should be interpreted with caution. It may either reflect the adaptive gut colonization in hosts or result from the environmental contamination during field sampling. Our results suggested that *Acinetobacter*, *Pseudomonas*, *Corynebacterium*, and *Halomonas* might be linked to the adaptation of Junggar Bactrian camels to wild grassland environments, although environmental interference cannot be fully excluded.

Gharechahi et al. (28) demonstrated that the forage rich in starch and polysaccharides promotes the colonization of *Prevotella* and *Streptococcus* in the gut of dromedary camels, while *Clostridium*, *Bacteroides*, and *Spirochaetes* are primarily responsible for degrading polysaccharides in the gut. Rabee et al. (29) and Gruninger et al. (30) fed Egyptian dromedary camels with three different types of forages and discovered that camels fed with fresh clover (high-quality forage) present greater diversity of gut microbiota, while those fed with wheat straw (low-quality forage) have higher pH, propionate, and butyrate levels in the gut, with *Prevotella* as the most abundant genus among the gut microbiota. Camels fed with a mixed diet exhibit higher abundance of bacteria and protozoa in the gut microbiota, among which the proportions of *Prevotella* and *Ruminococcus* are increased. These findings from dromedary camels provide valuable references for understanding the gut microbial adaptation of Bactrian camels. Consistent with the above observations, the results of this study indicated that *Acinetobacter*, *Pseudomonas*, *Corynebacterium*, and *Halomonas* were associated with the ability of Junggar Bactrian camels to adapt to wild grassland environments and effectively utilize natural feed resources. Meanwhile, *Escherichia* had the highest abundance in the gut of captive camels from southern Xinjiang, which is possibly correlated with the relatively confined, stable, and intensive feeding conditions under human management. The high abundance of *Escherichia* may increase the risk of diarrhea, and under certain conditions, *Escherichia* can serve as a pathogen to disrupt gut microbiota balance in animals and induce diarrhea in juvenile livestock (31). Given the high abundance of *Escherichia* in the gut of captive camels from southern Xinjiang and its potential pathogenicity, the monitoring and management of camels’ gut health should be strengthened, and diversified feed resources should be provided and supplemented with tender leaves.

As an enabler of plant fibre degradation in ruminants, the gut microbiota are rich in cellulolytic microbes and CAZymes (32). The results of this study indicated that GHs and GTs were the primary CAZy families responsible for degrading plant cellulose in forages by the gut microbiota in Bactrian camels. Higher abundance of GHs in Bactrian camels might reflect a stronger fiber degradation capability. Specifically, GHs can hydrolyze glycosidic bonds, thereby releasing monosaccharides or oligosaccharides for microbial utilization. GTs are responsible for transferring glycosyl groups to acceptor molecules, forming glycosidic bonds and participating in the synthesis and modification of polysaccharides, oligosaccharides, and glycoconjugates. During the degradation of plant cellulose, GTs may further promote microbial degradation and cellulose utilization by modifying or recombining the degradation products. Additionally, the gut microbiota in Bactrian camels reared under grazing had significantly increased abundance of eight CAZymes, among which CBM93 was an important component of cellulase. Besides, endowed with glycosyltransferase activity, GT53, GT85, GT87, and GT89 can utilize activated donors to form glycosidic bonds for specific glycosyl transfer reactions. PL5 is a polysaccharide lyase capable of degrading certain plant cell wall components or microbially produced polysaccharides. GH13 is involved in the degradation of plant cell wall, the microbial utilization of carbohydrates, and other functions. In summary, the large repertoire of GHs revealed in the rumen microbiome of the Bactrian camels indicates the strong fibre degradation function in the rumen of Bactrian camels.

Gut microbiota in animals plays an indispensable role in digestion, nutrient absorption, growth, production performance, and immune function (33). A healthy gut microbiota in lactating camels helps improve the quality and yield of milk. For juvenile camels with diarrhea, restoring the balance of gut microbiota is the key to treating diarrhea and enhancing immunity. Therefore, appropriate feeding strategies and nutritional regimes are crucial for maintaining the health of gut microbiota in camels. However, there is currently a lack of studies on the correlation between gut microbiota and nutritional requirements and disease resistance in Bactrian camels due to their high breeding costs, unique physiological structure, and the extreme difficulty in collecting gut fluid. Given that the limited sample size of this study might constrain the generalizability of the findings, and that the data were merely collected in a single season, future research should be conducted to investigate the succession patterns and functions of gut microbiota in various breeds of Bactrian camels across different regions. Artificial interventions can also be adopted to reduce the proportion of pathogens in the digestive tract and increase the diversity of probiotics to uncover potential relationships between gut microbiota variations and Bactrian camels, thus providing foundational data for research on breeding pattern and nutritional metabolism of Bactrian camels from various regions of China. Nevertheless, there are several notable limitations in the present study. Variations in geographical location, climate, diet and breeding patterns exist concurrently between the sampling sites in northern and southern Xinjiang. Consequently, the observed intergroup differences in gut microbiota in Bactrian camels cannot be attributed to breeding patterns alone. Therefore, it is necessary to further control the feeding trials under consistent environmental conditions to disentangle the independent effects of breeding systems and other environmental factors on gut microbiota.

## 5 Conclusion

The diversity and abundance of gut microbiota were greater in lactating camels reared under grazing in northern Xinjiang than those raised by barn feeding in southern Xinjiang, as well as greater in adult lactating camels than in juvenile camels. The halophilic and crude fiber-degrading microorganisms occupy a dominant position in the gastrointestinal tract of adult lactating camels raised under free-range grazing in northern Xinjiang. *Escherichia* and *Prevotella* are present with the highest abundance in the gastrointestinal tract of Bactrian camels raised by barn feeding in southern Xinjiang. The enhanced abundance of these taxa is potentially correlated with the altered homeostasis of gut microbiota, a state frequently associated with the increased opportunities for pathogens to attach to the intestinal epithelium and the raised risks of gastrointestinal disorders. The relative abundance of *Bifidobacterium* and *Lactobacillus* in the intestines of juvenile camels is significantly higher than that in adult lactating camels, indicating that these probiotics are critical players in nutrient absorption and immune defense during the development of the gut microbial ecosystem. The findings of this study demonstrate that geographical location, climate, and breeding patterns are important influencing factors for the composition and function of gut microbiota in camels. For Bactrian camels under barn feeding in southern Xinjiang, increasing the diversity of feed resource and adding tender forage may serve as potential optimization strategies. In practical camel husbandry, herders should monitor the digestive and metabolic status and immune performance in camels. Exogenous additives such as probiotics may reduce the relative abundance of intestinal pathogens, enrich beneficial microbes and improve intestinal health. Nevertheless, targeted verification should be further performed to support their practical application.

## Data Availability

The datasets presented in this study can be found in online repositories. The names of the repository/repositories and accession number(s) can be found in the article/Supplementary material.
